# Identifying transdiagnostic traumatic stress reactions in U.S. military veterans: A nationally representative study

**DOI:** 10.1002/jts.23119

**Published:** 2024-12-16

**Authors:** Cameron P. Pugach, Shane W. Adams, Blair E. Wisco, Robert H. Pietrzak

**Affiliations:** ^1^ Department of Psychology University of North Carolina at Greensboro Greensboro North Carolina USA; ^2^ Department of Neurosurgery Stanford University School of Medicine Palo Alto California USA; ^3^ Polytrauma System of Care VA Palo Alto Health Care System Palo Alto California USA; ^4^ National Center for PTSD, Clinical Neurosciences Division VA Connecticut Healthcare System Orange Connecticut USA; ^5^ Department of Psychiatry Yale School of Medicine West Haven Connecticut USA; ^6^ Department of Social and Behavioral Sciences Yale School of Public Health New Haven Connecticut USA

## Abstract

Traumatic stress reactions (TSRs) exist on a continuum that includes posttraumatic stress disorder (PTSD), highly comorbid psychopathology, and resilience, highlighting the need for comprehensive and integrative approaches capable of capturing the full spectrum of heterogeneous reactions. Here, we used a transdiagnostic and multidimensional method to characterize clinical phenotypes of TSRs in a nationally representative sample of U.S. military veterans. The Middle‐Out Approach was used to evaluate self‐reported PTSD, generalized anxiety, major depressive symptoms, and physical and mental functioning to identify discrete latent classes of TSRs and their demographic, military and trauma history, and psychosocial correlates. Cross‐sectional data were analyzed from 3,727 U.S. veterans who participated in the National Health and Resilience in Veterans Study. Latent class analysis identified five classes of veterans: low TSR (61.3%), anxious/depressive (16.6%), avoidant arousal (9.2%), dysphoric arousal (8.2%), and high TSR (4.7%). Veterans in the dysphoric arousal and high TSR classes demonstrated lower functioning than other classes, which showed similar levels of moderate‐to‐high functioning despite symptom differences. Classes distinguished between resilience to PTSD symptoms versus resilience to all symptoms and functioning domains and were differentially associated with demographic characteristics, trauma and military histories, and psychosocial characteristics. The results suggest that veterans exhibit different clinical phenotypes of TSRs, which may help inform etiology, diagnostic subtypes, and personalized treatment. Further, although most veterans with psychopathology experience functional impairment, a sizable subset demonstrates high functioning despite psychopathology symptoms.

## Identifying transdiagnostic traumatic stress reactions in U.S. military veterans: A nationally representative study

Veterans exposed to potentially traumatic events (PTEs) report a continuum of posttraumatic sequelae, ranging from adverse to resilient reactions. There is considerable interest in identifying where veterans fall along this continuum and the factors associated with these reactions (Galatzer‐Levy et al., 2018; Infurna & Luthar, 2018). Research on adverse traumatic stress reactions (TSRs) has largely focused on disorders, emphasizing discrete conditions such as posttraumatic stress disorder (PTSD) and related pathologies like anxiety and depression (Wisco et al., 2014) or symptom composites incapable of fully capturing the heterogeneity in clinical presentations of trauma‐related psychopathology (Adams et al., 2023; Hawn et al., 2022; Levin‐Aspenson & Greene, 2024). Research on resilience—defined as an outcome of successful adaptation to PTEs (Zautra et al., 2008)—has often used an outcome‐focused framework to identify trauma survivors who exhibit few or no symptoms of psychopathology (Bonanno et al., 2023). Using a narrow focus on symptoms to define resilience may be problematic, however, because indicators of negative functioning do not reflect adaptation across multiple life domains (Southwick et al., 2014). The present study sought to address these limitations by adopting a transdiagnostic and multidimensional approach to characterize veterans’ unique TSRs across the full continuum of trauma sequelae, ranging from adversity to resilience, and to identify correlates associated with these reactions.

## Transdiagnostic trauma‐related psychopathology presentations

PTSD has evolved into one of the most heterogeneous disorders in the *Diagnostic and Statistical Manual of Mental Disorders* (5th ed., text. rev.; *DSM‐5‐TR*; American Psychiatric Association [APA], 2022; Bryant et al., 2023; Galatzer‐Levy & Bryant, 2013). Veterans exposed to PTEs differ markedly in PTSD symptom severity (e.g., no symptoms, subthreshold symptoms, threshold symptoms for a full diagnosis; Klein et al., 2024) and symptom presentation. Indeed, there are well over 600,000 possible ways to meet the criteria for PTSD (Galatzer‐Levy & Bryant, 2013), with unique symptom presentations being the rule rather than the exception (Bryant et al., 2023). Several clinically and empirically meaningful PTSD subtypes that carry merit have been put forth (e.g., dysphoric arousal, threat; Adams et al., 2019). Further, diagnostic comorbidity occurs in an estimated 90% of people with PTSD (Koenen et al., 2008), with anxiety and depression being the most common in veterans (Wisco et al., 2014). Notably, not every individual will experience all PTSD symptoms, and people may differ in their presentations of PTSD and comorbid anxiety and depressive symptoms (e.g., Flory & Yehuda, 2015; Klein et al., 2024). Relying on PTSD symptom composite scores as the sole index of TSRs might, thus, exclude other essential clinical presentations.

The dimensional nature of symptom reactions, heterogeneity in PTSD symptom presentations, and high rates of psychiatric comorbidities have fueled calls for approaches capable of capturing transdiagnostic and person‐centered psychopathology presentations across the full range of TSRs (Adams et al., 2023; Hawn et al., 2022; Levin‐Aspenson & Greene, 2024). This shift toward identifying person‐centered TSRs is particularly important when considering issues of treatment nonresponsiveness and drop‐out in veterans who receive first‐line, trauma‐focused psychotherapies (Steenkamp et al., 2020). Indeed, a “one size fits all” approach may fail to attend to the complexity and heterogeneity of TSRs (Herzog & Kaiser 2022). For example, among the many barriers to engaging with trauma‐focused treatments identified by veterans is a lack of attention to individuals’ unique needs and presentations (Kehle‐Forbes et al., 2022). A shift toward identifying person‐centered TSRs could ultimately help improve treatment access, engagement, and retention by informing the development, selection, and sequencing of tailored interventions to address the unique concerns of these clinical presentations.

## Integrating adaptive TSRs

Although transdiagnostic approaches permit the identification of person‐centered and clinically meaningful presentations of trauma‐related psychopathology, a narrow focus on symptoms limits understanding and treating the full spectrum of TSRs. There is also considerable interest in identifying resilient responses to PTEs among adults and the factors that may help promote them (Bonnano et al., 2023; Galatzer‐Levy et al., 2018). Although several approaches to operationalizing resilience exist (Troy et al., 2023), a common “outcome‐focused” approach used in longitudinal studies is the absence of psychopathology symptoms during and after PTE exposure. This has led to the conclusion that most adults exposed to PTEs, including veterans, are resilient (e.g., approximately 66%; Galatzer‐Levy et al., 2018). One criticism levied against this conclusion is the prominent unidimensional focus on psychopathological symptoms (Infurna & Luthar, 2018). The absence of symptoms does not necessarily mean one is doing well in other domains, nor does the presence of psychological symptoms necessarily preclude positive functioning in other areas (Infurna & Luthar, 2017). Integrating indicators of adverse and adaptive responding has thus been proposed as a method to better characterize TSRs (Infurna & Luthar, 2018; Southwick et al., 2014).

Health‐related functioning is one well‐studied indicator of positive adaptation among veterans. Health‐related functioning (henceforth referred to as *functioning*), reflects one's self‐perceived physical and mental functioning (Ware et al., 2001). Lower mental and physical functioning is consistently associated with PTSD (Rodriguez et al., 2012) and comorbid mood and anxiety disorders (Rapaport et al., 2005). Despite these negative associations, empirical and clinical evidence suggest variability in the association between trauma‐related psychopathology and adaptive functioning among veterans (Gower et al., 2024). For example, some veterans might experience higher levels of functioning despite their symptoms, possibly reflecting “learning to live with” chronic PTSD or a capacity to push forward despite adversity (Southwick et al., 2014). Thus, integrating adaptive measures of functioning into person‐centered TSRs has the potential to (a) clarify resilience across multiple dimensions, (b) identify how heterogeneous psychopathology symptom presentations are differentially related to functioning, (c) uncover complex TSRs that are difficult to study at the variable level (e.g., a subset of veterans who report high functioning despite psychopathology), and (d) quantify risk and protective factors associated with these unique TSRs.

## Phenotypes of TSRs: The Middle‐Out Approach

The Middle‐Out Approach represents a novel method for conceptualizing, assessing, and analyzing person‐centered clinical presentations or phenotypes of TSRs (Adams et al., 2023). This approach is an integrative, contextually informed framework that involves the use of (a) transdiagnostic and multidimensional data sources that cut across psychiatric disorders and other biopsychosocial domains and (b) a combination of person‐centered (e.g., mixture models, such as latent class analysis [LCA]) and variable‐centered (e.g., logistic regression) approaches to identifying and helping support, respectively, theoretically informed clinical phenotypes and their correlates in heterogeneous samples. Within the Middle‐Out Approach, “middle‐order” clinical phenotypes are discrete, latent, person‐centered profiles reflecting unique and often transdiagnostic latent constructs that are less constrained by “higher‐order” diagnoses and “lower‐order” items or symptoms. The Middle‐Out Approach, thus, permits a holistic and empirical evaluation of TSRs, grounded in theory, to identify person‐centered presentations and inform clinical decision‐making beyond information that would be gained from purely diagnostic or symptom perspectives (e.g., PTSD diagnosis).

## The current study

This study sought to apply the Middle‐Out Approach to identify clinical phenotypes of TSRs in a nationally representative veteran sample by evaluating a range of psychopathological symptoms across three psychiatric disorders (i.e., PTSD, generalized anxiety, major depression) and two forms of adaptive functioning (i.e., physical, mental) using LCA. Measures of total lifetime trauma exposure, time since trauma, and mental health treatment history were also included in the LCA to determine if phenotypes were demarcated by cumulative trauma load, natural opportunity for recovery, or past treatment. Finally, theoretically relevant risk and protective factors, including demographic characteristics, trauma and military histories, and psychosocial characteristics (i.e., protective psychological traits, social connectedness, and loneliness) were selected to examine correlates of phenotypes classification, inform theory, and identify potentially modifiable targets for intervention.

We hypothesized that two well‐founded phenotypes would emerge: a *high TSR* phenotype characterized by high levels of severity across all symptoms and low functioning and a *low TSR* phenotype consisting of low severity across all symptoms and high functioning (Galatzer‐Levy et al., 2018). We further expected that the LCA would also yield unique presentations that differed in both the type and severity of concerns. We expected to see a phenotype marked by low levels of PTSD symptoms and higher levels of depressive and anxiety symptoms, a phenotype characterized by high PTSD symptom levels and lower depressive and anxiety symptom levels, and one or more phenotypes differentiated by transdiagnostic domains (e.g., arousal, dysphoria). Of particular interest was how physical and mental functioning manifested across potential phenotypes and the unique symptom constellations associated with differences in functioning. Finally, we hypothesized that demographic, trauma and military histories, and psychosocial characteristics would differ between phenotypes.

## METHOD

### Participants

Data were drawn from the National Health and Resilience in Veterans Study (NHRVS; Pietrzak et al., 2021), which surveyed a nationally representative sample of U.S. veterans between November 2019 and March 2020, with a median completion date of November 21, 2021. Participants were 4,069 U.S. veterans who anonymously completed an online survey. Of the 4,069 veterans, 342 did not report their PTSD symptoms and were excluded from the analyses, leaving a final sample size of 3,727. Table 1 shows the final sample demographic, military, trauma, and clinical characteristics.

**TABLE 1:** Demographic, trauma and military history, and clinical characteristics of the final analytic sample

Variable	Weighted *M*	Weighted *SD*	*n*	Weighted %
**Demographic characteristics**				
Age (years)	61.7	15.5		
Sex				
Male			3,247	89.7
Female			480	12.88
Race/ethnicity				
White, non‐Hispanic			3,034	78.1
Black, non‐Hispanic			259	10.6
Hispanic			295	7.0
Other, non‐Hispanic			46	2.7
≥ 2 races, non‐Hispanic			93	1.6
Household income (USD)				
< $10,000			77	2.7
$10,000–$19,999			160	5.1
$20,000–$39,999			633	16.7
$40,000–$59,999			672	15.9
$60,000–$99,999			1,203	26.4
$100,000–$199,999			863	27.3
≥ $200,000			119	5.9
Educational attainment				
Less than high school			22	1.3
High school			448	27.3
Some college			1,563	37.7
Bachelor's degree or higher			1,694	33.7
Currently employed			1,503	48.9
**Military and trauma history**				
Combat veteran			1,269	35.7
Index trauma type^a^				
Disaster/accident			1,464	42.5
Illness/injury			1,038	29.8
Interpersonal violence			474	14.4
Combat/captivity			425	12.6
Injury/harm/death to other			33	0.8
**Clinical characteristics** ^b^				
Probable PTSD			167	5.6
PTSD symptoms (PCL‐5)	8.33	13.4		
Depressive symptoms (PHQ‐4)	0.72	1.32		
Anxiety symptoms (PHQ‐4)	0.68	1.27		

*Note*: *N* = 3,727. PTSD = posttraumatic stress disorder; PCL‐5 = PTSD Checklist for *DSM‐5*; PHQ‐4 = four‐item Patient Health Questionnaire.

^a^ *n* = 293 participants did not report an index trauma type Type on LEC‐5

^b^ PTSD, depressive symptoms, and anxiety symptoms were calculated continuously using the PCL‐5 and PHQ‐4. Probable PTSD was calculated using the *Diagnostic and Statistical Manual of Mental Disorders* (5th ed.; *DSM*‐*5*) criteria.

### Procedure

The NHRVS sample was drawn from KnowledgePanel, a survey research panel of over 50,000 U.S. households maintained by the survey research firm Ipsos. KnowledgePanel is an online, probability‐based, nonvolunteer survey panel of a nationally representative sample of noninstitutionalized U.S. adults covering about 98% of U.S. households. Panel members were recruited through national random samples, originally by telephone, then primarily by postal mail. A total of 7,860 veterans were invited to participate in the baseline survey, with 4,069 (51.8%) completing the survey. Ipsos statisticians computed poststratification weights using benchmark distributions of U.S. military veterans from the most contemporaneous 2019 Current Veteran Population Supplemental Survey of the U.S. Census Bureau's American Community Survey (e.g., age, sex, race, ethnicity; U.S. Census Bureau, 2019). Poststratification weights allow for the generalizability of results to the general U.S. veteran population. Veterans provided electronic informed consent, and all procedures were approved by the Human Subjects Committee of the VA Connecticut Healthcare System.

### Measures

#### Phenotyping characteristics (LCA indicators)

**Lifetime trauma exposure**. The Life Events Checklist for *DSM‐5* (LEC‐5; Weathers, Blake, et al., 2013b) is a 17‐item questionnaire used to assess lifetime exposure to 16 PTEs known to predict PTSD (e.g., sexual assault, combat exposure), plus an additional item to capture unlisted stressful experiences. For each endorsed event, veterans indicated their level of exposure (i.e., “happened to me,” “witnessed it,” “learning about it,” “part of my job”). We created a composite of lifetime trauma exposure by summing the number of exposures across all event types. Veterans who reported exposure to multiple events also identified their index (i.e., “worst”) event and were asked to use this event as a reference when rating PTSD symptoms. Time since trauma exposure was measured as the age of index event onset from current age.

**PTSD symptoms**. The PTSD Checklist for *DSM‐5* (PCL‐5; Weathers, Litz, et al., 2013) is a 20‐item self‐report measure of *DSM‐5* PTSD symptoms. Veterans rated each PTSD symptom on a 5‐point Likert scale ranging from 0 (*not at all*) to 4 (*extremely*), using the index event identified on the LEC‐5 as a reference. In reporting descriptives, we summed PCL‐5 items to create a total PTSD symptom composite ranging from 0 to 80, with a cutoff score of 33 indicating a probable PTSD diagnosis (Bovin et al., 2016). To aid LCA model fit, PCL‐5 items were dichotomized as moderate severity or above (i.e., a score of 2 or higher), consistent with clinical interview procedures (Weathers, Blake, et al., et al., 2013a). In this sample, internal consistency of the PCL‐5 was excellent, Cronbach's α = .96.

**Depressive and anxiety symptoms**. The Patient Health Questionnaire–4 (PHQ‐4; Kroenke et al., 2009) is a four‐item self‐report measure of the cardinal symptoms of anxiety (i.e., anxiety and worry) and depression (i.e., depressed mood and anhedonia) experienced over the past 2 weeks. Participants rated each item on a 4‐point Likert scale ranging from 0 (*not at all*) to 3 (*nearly every day*), with two items comprising each subscale (i.e., Depression and Anxiety). For descriptive purposes, we summed the items on each subscale, with total scores of 3 or higher indicating the presence of anxiety or depression (Kroenke et al., 2009). To aid LCA model fit and consistent with past research (Teymoori et al., 2020), PHQ‐4 items were dichotomized as experiencing symptoms several days or more (i.e., a score of 1 or higher) to reflect the clinical presence of each symptom. Internal consistency was good for the Anxiety subscale, *r* = .86, and adequate for the Depression subscale, *r* = .76.

**Physical and mental functioning**. The Short Form–8 Health Survey (The SF‐8; Ware et al., 2001) was used to assess physical and mental health functioning. The SF‐8 consists of two subscales with four items each: the Physical Component Summary (PCS), which assesses physical functioning, and the Mental Component Summary (MCS), which assesses mental functioning. Veterans were asked to rate each item relative to their past‐month experience on a scale of 0 to 100, and scores were calculated by averaging item scores for each subscale, with higher scores indicating better functioning. The SF‐8 uses norm‐based scoring methods from studies of the general U.S. population (*M* = 50, *SD* = 10). We analyzed functioning scores continuously at the subscale level to represent physical (PCS) and mental (MCS) functioning. In the present sample, internal consistency was good for both subscales, PCS: Cronbach's α = .87, MCS: Cronbach's α = .81.

**Mental health treatment history**. One item, “Have you ever received mental health treatment (e.g., prescription medication or psychotherapy) for a psychiatric or emotional problem?,” was used to assess lifetime mental health treatment history. Veterans rated this item dichotomously (0 = “no,” 1 = “yes”). We included this item to examine if the hypothesized low TSR phenotype was an artifact of having received previous mental health treatment.

#### Correlates of phenotype classification (auxiliary variables)

**Demographic characteristics**. Demographic characteristics included age, sex, race/ethnicity, educational attainment, annual household income, and current employment status.

**Trauma and military histories**. A categorical variable representing index trauma type was created based on each veteran's index event as indicated on the LEC‐5. Events were coded into one of two categories: interpersonal index events (e.g., interpersonal violence) and noninterpersonal index events (e.g., disaster, accidents, illness or injury). A single item, “Have you ever served in a combat or war zone?” was used to assess combat veteran status. All veterans who answered “yes” to this item were classified as combat veterans in the analyses.

**Protective psychosocial characteristics and social connectedness**. As detailed elsewhere (Pietrzak et al., 2021), factor analysis was used to reduce several scales included in the NHRVS survey into two factors representing protective psychosocial characteristics and social connectedness. The protective psychosocial characteristics factor included measures of purpose in life, grit, resilience, optimism, gratitude, curiosity, and perceived community integration. The social connectedness factor included measures of perceived social support, structural social support, and attachment style. Factor scores were used in the analyses. See Supplementary Table S1 for details of these measures.

**Loneliness**. Loneliness was measured using a three‐item abbreviated version of the UCLA Loneliness Scale (Hughes et al., 2004). Items were rated on a 3‐point Likert scale ranging from 1 (*hardly ever*) to 3 (*often*). Scores range from 3 to 9, with higher scores indicating higher degrees of loneliness. In this sample, internal consistency for the UCLA Loneliness Scale was good, Cronbach's α = .87.

### Data analysis

We used LCA to identify homogenous clinical phenotypes of TSRs within the larger, more heterogeneous sample of veterans exposed to PTEs. Full information maximum likelihood estimation was used to compare one‐class to six‐class models and identify the best‐fitting model. Lower fit indices, including the Bayesian information criterion (BIC), sample‐size adjusted BIC (ssBIC), and Akaike information criterion (AIC), in addition to higher entropy values, were used to indicate better model fit. The Lo–Mendell–Rubin adjusted likelihood ratio test (LMR‐LRT) and bootstrapped likelihood ratio test (BLRT) were used to indicate whether subsequent models demonstrated significantly (*p* < .05) better model fit than previous models (Nylund‐Gibson & Choi, 2018). A combination of these indices and several other considerations (i.e., having more than 5% of the total sample in each class, high posterior probabilities of correct classifications, theoretical coherence, parsimony, and clinical significance) were taken into account when choosing the final model (Nylund‐Gibson & Choi, 2018; Nylund et al., 2007). To address issues of conditional independence between correlated indicators, we relaxed the local independence assumption by estimating parameters considering the residual associations between indicators and a higher number of random starts (Visser & Depaoli, 2022).

An iterative approach to model estimation was implemented. We first entered the 20 PCL‐5 items to identify an initial model, then added the four PHQ‐4 items to supplement or modify the previous model to produce a final unconditional model capturing person‐centered symptom presentations. Unconditional models were compared by first entering symptom data categorically using LCA versus continuously using latent profile analysis (LPA). Models were evaluated based on model fit, model convergence and cohesion among the larger set of variables, and the theoretical and clinical significance of the resulting classifications. Ultimately, an unconditional LCA model using dichotomized PCL‐5 and PHQ‐4 items provided an optimal model solution that was more clinically meaningful in demonstrating differences in both symptom severity and the type of symptoms reported (for a description of and comparison with the best‐fitting LPA model, see Supplementary Table S2). The two functioning variables (i.e., PCS and MCS), lifetime trauma exposure, time since the index traumatic event, and mental health treatment history were then entered using a similar iterative process to obtain a final conditional model. Functioning, lifetime trauma exposure, and time since trauma were included as continuous summed variables, and mental health treatment history utilization was included as a dichotomous variable using the same methodological scrutiny as previously described. Once an optimal conditional model was identified, the three‐step approach (Asparouhov & Muthén, 2013) was used to nest a multinomial logistic regression into the LCA to examine the associations between latent classes and auxiliary variables of interest (e.g., loneliness, social connectedness).

## RESULTS

### Phenotypes of trauma‐related psychopathology and functioning

Supplementary Table S3 presents the descriptive statistics for all LCA indicators and correlates. LCAs were constructed using an iterative process, first identifying the optimal model for the 20 categorical PCL‐5 items, then introducing the 4 categorical PHQ items to the previous model until a final unconditional model was identified evaluating PCL‐5 and PHQ‐4 items concurrently. The two SF‐8 total scores reflecting physical and mental adaptive functioning were then entered with trauma exposure, time since trauma, and treatment history into a final conditional model, which was used for further analyses. Although PTSD symptoms, anxiety symptoms, depressive symptoms, and functioning indicators were evaluated within the same model, the results are illustrated in separate figures for ease of presentation and interpretation.

A five‐class unconditional model demonstrated optimal fit when examining PTSD symptoms, depressive symptoms, and anxiety symptoms (see Supplementary Table S2). The average posterior probability of participants being correctly (87%–98%) and incorrectly (0%–4%) classified indicated high classification accuracy and specificity. When adding functioning, lifetime trauma exposure, time since trauma, and mental health treatment utilization history to a conditional model, fit indices improved further (i.e., 90.3%–96.8% probability of correct classification, 0%–4.6% probability of incorrect classification), suggesting the final five‐class solution was most suitable for interpretation (see item estimates in Supplementary Table S4.) When adding conditional variables, the proportion of veterans in each class shifted by less than 0.5%, suggesting that other indicators were more critical for classification than lifetime PTE exposure, time since trauma, or treatment history.

**FIGURE 1:**
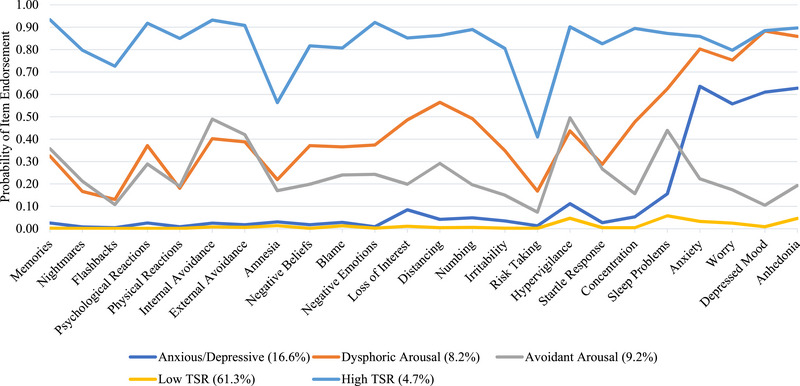
Illustration of latent classes using item estimates from past‐month PTSD Checklist for DSM‐5 (PCL‐5) and Patient Health Questionnaire–4 (PHQ‐4) administrations *Note*: PTSD = posttraumatic stress disorder; TSR = traumatic stress reactions.

The final included classes were: *low TSR* (*n* = 2,400, 61.3%), characterized by a low probability of endorsing most PTSD symptoms, anxiety symptoms, and depressive symptoms along with a high level of functioning; *anxious/depressive* (*n* = 590, 16.6%), characterized by low PTSD symptoms, moderate anxiety and depressive symptoms, and moderate functioning; *avoidant arousal* (*n* = 343, 9.2%), characterized by moderate avoidance and hyperarousal PTSD symptoms, low anxiety and depressive symptoms, and high functioning; *dysphoric arousal* (*n* = 257, 8.2%), characterized by moderate avoidance, negative alterations in cognition and mood, and hyperarousal PTSD symptoms along with high anxiety and depressive symptoms and low functioning; and *high TSR* (*n* = 137, 4.7%), characterized by high PTSD symptoms, anxiety symptoms, and depressive symptoms and low functioning (see Figure 1).

**FIGURE 2:**
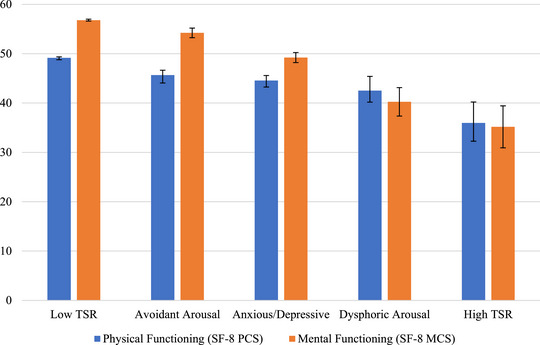
Estimated means for latent class scores on current physical and mental functioning *Note*: The Physical Component Score (PCS) and Mental Component Score (MCS) subscales of the Short Form–8 (SF‐8) use norm‐based scoring methods derived from studies of the general U.S. population, with a mean score of 50 and a standard deviation of 10. Higher scores indicate better functioning. Error bars reflect 95% confidence intervals. TSR = traumatic stress reactions.

Veterans in the low TSR class reported the highest mean levels of physical and mental functioning (0.5 standard deviations above the mean; Figure 2). Veterans in the avoidant arousal class and, to a lesser extent, the anxious/depressive class, also reported higher levels of functioning despite differences in symptoms, with scores ranging from ‐0.5 to 0.5 standard deviations from the mean. In these three classes, within‐class comparisons showed that veterans reported higher mental functioning than physical functioning. Alternatively, veterans in the high TSR class (1.5 standard deviations below the mean) and the dysphoric arousal class (1.0 to 0.8 standard deviations below the mean) were characterized by the lowest levels of physical and mental functioning. Unlike the other classes, within‐class comparisons suggested that veterans in the high TSR and dysphoric arousal classes reported similar levels of mental and physical functioning.

### Correlates of identified phenotypes

A three‐step approach was used to evaluate demographic characteristics, trauma and military histories, and psychosocial characteristics of interest (i.e., psychosocial protective factors, social connectedness, and loneliness) as correlates of class membership (Table 2). The low TSR class was used as the reference class. Veterans in the avoidant arousal class were more likely to be female, adjusted odds ratio (a*OR*) = 2.38, and a veteran of color, a*OR* = 1.86. Veterans in the anxious/depressive class were more likely to be unemployed, a*OR =* 1.43, and a veteran of color, a*OR =* 1.62. Veterans in the dysphoric arousal class were more likely to be unemployed, a*OR =* 1.83. Veterans in the high TSR class were more likely to be female, a*OR =* 2.43; unemployed, a*OR =* 3.80; and a veteran of color, a*OR =* 3.01.

**TABLE 2:** Three‐step approach using multinomial logistic regression to identify correlates of latent class analysis (LCA) class membership

Correlate^a^	Anxious/depressive		Avoidant arousal		Dysphoric arousal		High TSR	
	a*OR*	95% CI	a*OR*	95% CI	a*OR*	95% CI	a*OR*	95% CI
**Demographic characteristics**								
Age	0.98^***^	[0.97, 0.99]	0.99^*^	[0.97, 1.00]	0.95^***^	[0.94, 0.97]	0.94^***^	[0.92, 0.96]
Sex								
Male	Ref.							
Female	1.44	[0.99, 2.08]	2.38^***^	[1.60, 3.55]	1.53	[0.92, 2.54]	2.43^**^	[1.29, 4.60]
Race/ethnicity^b^								
White, non‐Hispanic	Ref.							
Veteran of color	1.62^**^	[1.19, 2.20]	1.86^***^	[1.33, 2.62]	1.27	[0.77, 2.12]	3.01^***^	[1.77, 5.10]
Employment status								
Currently employed	Ref.							
Currently unemployed	1.43^*^	[1.06, 1.92]	1.25	[0.87, 1.80]	1.83^**^	[1.18, 2.84]	3.80^***^	[2.12, 6.81]
**Trauma and military history**								
Combat veteran	1.01	[0.98, 1.03]	1.90^***^	[1.42, 2.54]	1.00	[0.96, 1.03]	2.95^***^	[1.89, 4.60]
Index trauma type								
Noninterpersonal event	Ref.							
Interpersonal event	1.09	[0.74, 1.61]	2.18^***^	[1.52, 3.12]	1.36	[0.85, 2.20]	1.53	[0.85, 2.78]
**Psychosocial factors**								
Protective psychosocial factors	0.44^***^	[0.37, 0.51]	0.84	[0.68, 1.05]	0.33^***^	[0.26, 0.41]	0.23^***^	[0.18, 0.31]
Social connectedness	0.83^*^	[0.71, 0.98]	0.77^*^	[0.61, 0.96]	0.69^**^	[0.52, 0.92]	0.61^**^	[0.44, 0.85]
Loneliness	1.46^***^	[1.33, 1.60]	1.34^***^	[1.21, 1.49]	1.90^***^	[1.67, 2.17]	1.70^***^	[1.47, 1.96]

*Note*: The reference category (Ref.) was low traumatic stress reactions (TSR). See Supplementary Table S5 for correlate descriptives within each class. See Supplementary Table S6 for a breakdown of class membership by race/ethnicity. a*OR* = adjusted odds ratio; CI = confidence interval.

^a^ *n =* 314 (8.4%) participants were excluded from the analyses due to missing data on correlates. Missing data within each class were as follows: low TSR: *n* = 160, 6.7%; anxious/depressive: *n* = 52, 8.7%; avoidant arousal: *n* = 40, 11.6%; dysphoric arousal: *n* = 40, 15.4%; high TSR: *n* = 22.

^b^ Dichotomized due to low cell sizes

*p < .05. **p < .01. *** *p* <.001.

Veterans who served in a combat or war zone had increased odds of being in the avoidant arousal class, a*OR =* 1.90, or the high TSR class, a*OR =* 2.95, but not the anxious/depressive or dysphoric arousal classes. Veterans who reported an interpersonal index traumatic event were more likely to be in the avoidant arousal class, a*OR =* 2.18, but not other classes.

Finally, veterans with fewer protective psychosocial characteristics were more likely to be in the anxious/depressive, a*OR =* 2.27; dysphoric arousal, a*OR =* 3.03; and high TSR classes, a*OR =* 4.35. Note that adjusted odds ratios less than 1.0 were divided from 1 for ease of interpretation (e.g., 1/0.44 = 2.27). Veterans in the avoidant arousal and low TSR classes reported similar protective psychosocial characteristics. Veterans with lower social connectedness were more likely to be in all classes compared to the low TSR class, anxious/depressive: a*OR =* 1.20; avoidant arousal: a*OR =* 1.30; dysphoric arousal: a*OR =* 1.45; high TSR: a*OR =* 1.64. Loneliness was similarly associated with increased odds of being in all classes, a*OR*s *=* 1.34–1.90, but most strongly associated with the dysphoric arousal class.

## DISCUSSION

This study sought to identify person‐centered clinical phenotypes of TSRs in a nationally representative sample of U.S. veterans. Consistent with our hypotheses, several distinct presentations emerged, differing in both symptom composition and functioning. The results suggested five unique clinical phenotypes of trauma‐related risk and resilience in U.S. veterans: low TSR (61.3%), anxious/depressive (16.6%), avoidant arousal (9.2%), dysphoric arousal (8.2%), and high TSR (4.7%). Veterans who were female, unemployed, and veterans of color generally had a higher risk of more symptomatic phenotypes, as were veterans with a combat history, fewer psychosocial protective characteristics, less social connectedness, and higher levels of loneliness. We discuss the interpretation and conceptualization of these resilient and symptomatic phenotypes, implications for classifying and treating TSRs, and limitations and future directions below.

Different conceptualizations of resilience exist in mental health research, notably regarding whether resilience is treated as a trait, process, or outcome (Southwick et al., 2014). Mental health research in trauma‐exposed adults often conceptualizes resilience as an outcome (Troy et al., 2023). For example, resilience has been described as stable, low levels of psychological symptoms at or near normal levels (Galatzer‐Levy et al., 2018); as the absence of PTSD symptoms specifically (Bonanno & Mancini, 2012); or as a multidimensional phenomenon that reflects doing well in some domains while struggling in others (Infurna & Luthar, 2018). In the current study, phenotypes reflecting each of these forms of resilience were identified by varying combinations of low probabilities of symptom endorsement and higher functioning.

In the low TSR group, most veterans demonstrated low global symptoms of PTSD, anxiety, and depression, along with higher functioning, despite reporting similar levels of trauma exposure as the other classes. This low TSR class resembled the two thirds of adults in longitudinal research who have been found to demonstrate a resilience trajectory of low symptoms following PTE exposure (Galatzer‐Levy et al., 2018).

Participants in the anxious/depressive class demonstrated what could be termed resilience when considering PTSD alone (e.g., Bonanno & Mancini, 2012). However, these veterans also reported higher anxiety and depressive symptom levels with moderate levels of functioning, suggesting the presence of risk, rather than resilience, in some domains. Despite similar reports of trauma exposure and type, veterans in the anxious/depressive class reported more psychosocial disadvantage compared to those in the low TSR group (e.g., unemployed veterans of color who endorsed more loneliness and fewer protective psychological characteristics). Together, this profile suggests that the anxious/depressive phenotype represents a class of veterans struggling with adverse life circumstances, which could contribute to their current symptoms and functioning.

Veterans in the avoidant arousal class could be described as “functionally resilient” in that they reported functioning and psychosocial protective characteristics (e.g., trait resilience, purpose in life, optimism) similar to the low TSR phenotype, despite moderate levels of PTSD‐related avoidance, hypervigilance, and sleep disturbance symptoms. Veterans with the avoidant arousal phenotype, who were twice as likely to be female combat veterans of color who reported an interpersonal index trauma compared to those in the low TSR class, could reflect a subset of veterans who have learned to manage their symptoms to achieve high levels of functioning (Southwick et al., 2014). The avoidant arousal phenotype speaks to the promise of clinical interventions that target enhancing psychosocial factors (e.g., purpose in life, gratitude, community integration) and improving functioning in veterans with PTSD (Kaiser et al., 2024). Such targets are consistent with Veteran Health Affairs (VHA) Whole Health initiatives that aim to develop and disseminate interventions that enhance functioning and quality and life, particularly among veterans who struggle with trauma‐focused treatments (Kligler et al., 2022).

A small proportion of veterans reported high global levels of PTSD, depressive, and anxiety symptoms, along with low functioning, relative to the other classes. This high TSR class bears resemblance to chronic symptom trajectories shown in single‐outcome studies (Galatzer‐Levy et al., 2018), as well as multidimensional trajectories of “nonresilience” (Infurna & Luthar, 2017). Beyond this well‐established high TSR class, however, the results speak to the pernicious effects of dysphoria, hyperarousal, and globally elevated symptoms (Flory & Yehuda, 2015).

Dysphoric arousal is a distinct phenotype that largely represents PTSD symptoms of dysphoria and hyperarousal and has been identified across clinical, community, and national samples, suggesting the validity of this clinical phenotype of traumatic stress (Adams et al., 2019, 2024; Elhai & Palmieri, 2011). Here, we observed that social withdrawal, loss of interest, and emotional numbing PTSD symptoms—in concert with avoidance and hyperarousal PTSD symptoms and anxiety and depressive symptoms—comprised a distinct dysphoric arousal phenotype. Despite reporting fewer symptoms, veterans in the dysphoric arousal class had comparable levels of mental functioning, anxiety symptoms, and depressive symptoms relative to those in the high TSR group, which reflects comorbid PTSD, anxiety, and depression. In contrast, physical functioning was higher among veterans in the dysphoric arousal class than those in the high TSR class and comparable to those in the avoidant arousal and anxious/depressive classes. That mental functioning was especially low in the dysphoric arousal group aligns with research showing that dysphoria and arousal symptoms have a particularly adverse effect on global health trajectories and treatment prognosis (Adams & Allwood, 2024; Adams et al., 2019, 2024).

Veterans in the dysphoric arousal group also reported lower social connectedness and more loneliness compared to those in the low TSR group and had the highest odds of loneliness across all phenotypes, potentially reflecting the cumulative adverse impact of symptomatology and psychosocial disadvantage (e.g., unemployment, fewer protective factors). This result aligns with a general “loneliness model” (Hawkley & Cacioppo; 2010), which has been adapted to PTSD models and posits that trauma‐exposed individuals who perceive themselves as socially isolated tend to attend to more negative environmental stimuli and perceive the world as threatening, resulting in a chronic state of hyperarousal in service of protective behavior (Adams et al., 2024). Accordingly, social disconnection and loneliness in addition to social disadvantage may be implicated in the role that dysphoric arousal symptoms play in functional declines, adverse health outcomes, and poorer treatment prognosis. Such factors can help identify and target at‐risk veterans, who may have subthreshold PTSD symptoms, over and above the effects of combat exposure, which was not associated with the dysphoric arousal class in this sample. Evidence‐based treatments that target loneliness and social connectedness by improving relational functioning (e.g., alliance‐focused therapy, mentalization‐based therapy, VA social prescription programs) might help improve treatment prognosis for veterans with dysphoric arousal.

The avoidant arousal and dysphoric arousal phenotypes are consistent with PTSD conceptualizations from the World Health Organization's (WHO) *International Classification of Diseases and Related Health Problems* (*ICD‐11*; WHO, 2019) and the APA's (2022) *DSM‐5‐TR*, respectively. The avoidant arousal phenotype, akin to the seven‐symptom *ICD‐11* definition of PTSD, was characterized by intrusive, avoidance, and hyperarousal symptoms and shared low comorbidity with anxiety and depression. The dysphoric arousal and high TSR phenotypes, akin to the 20‐symptom *DSM‐5‐TR* definition of PTSD, were characterized by intrusive, avoidance, negative alterations in cognition and mood, and hyperarousal symptoms and shared high comorbidity with anxiety and depression.

Compared to the *DSM‐5‐TR*, the *ICD‐11* formulation of PTSD aims to achieve a simple definition less confounded by “nonspecific” PTSD symptoms that overlap with other disorders (Brewin, 2013). However, some research suggests that the *ICD‐11* operationalization of PTSD underidentifies individuals with PTSD while maintaining similar levels of comorbidity (Wisco et al., 2016). The results of this study suggest that an “either/or” approach will likely fail to adequately capture the heterogeneity in TSRs that exists between individuals. Instead, both the *ICD‐11* and *DSM‐5‐TR* conceptualizations of PTSD were identified as homogeneous phenotypes here, supporting their validity. Notably, the dysphoric arousal phenotype was differentiated from the high TSR phenotype, potentially suggesting replicated subtypes of *DSM‐5‐TR* criteria for PTSD that may help explain heterogeneous symptom presentations and guide targeted prevention and intervention efforts. Future work should examine the mechanisms underlying these phenotypes and explore whether existing treatments can be personalized to address these unique presentations.

The phenotypes identified here also have treatment implications. First, they support the use of comprehensive measurement‐based care for treatment selection. For example, had only PTSD symptoms been assessed, the resilient and anxious/depressive phenotypes may have clustered into a homogenous resilient class. Including anxiety and depressive symptoms and measures of functioning showed that although both classes demonstrated low PTSD symptom levels, the anxious/depressive class was distinguished by risk in other domains. Second, the same PTSD composite scores can reflect different phenotypic expressions of PTSD. Given work suggesting that dysphoric arousal symptoms are linked to worse health trajectories and treatment outcomes (Adams & Allwood, 2024; Adams et al., 2019, 2024), veterans with this presentation may benefit from alternative treatment approaches (e.g., stage‐based treatments, treatments targeting relational functioning) for increased engagement, retention, and success (Adams et al., 2024). Finally, the results suggest that high functioning can coexist with trauma‐related psychopathology, at least in some veterans. This finding provides empirical support for VHA Whole Health initiatives, which suggest that trauma‐exposed veterans can benefit from comprehensive health care approaches designed to promote well‐being (Kaiser et al., 2024; Kligler et al., 2022).

Despite its strengths, this study has some limitations. First, self‐report measures that assess psychopathology and functioning may be limited by response biases, low insight, demand characteristics, and social acceptability (Bovin & Marx, 2023). Clinical interviews are needed to confirm diagnostic status. Second, this sample reflects the demographic composition of the predominantly older, White, and male U.S. veteran population. This sample composition likely contributed to a ceiling effect for measures of physical and mental functioning and longer time since trauma. The findings may not generalize to other aspects of functioning (e.g., psychosocial functioning) or veteran subpopulations (e.g., younger veterans) in which trauma exposure might be more recent and the distribution of physical and mental functioning scores would likely cover the full range. Third, the cross‐sectional design precludes temporal inferences about the effects of PTE exposure and trauma reactions. Finally, the phenotypes modeled here largely fell under the internalizing dimension, and our measure of functional impairment shares some conceptual overlap with the symptoms assessed. Future research could expand the identification of phenotypes by including other symptom dimensions (e.g., externalizing symptoms) and measures of functioning with more symptom separation (e.g., relational measures).

Notwithstanding these limitations, results suggest that U.S. military veterans cluster into unique clinical phenotypes that represent discrete TSRs. Differential symptom types and severity levels, in addition to functioning, each played a role in differentiating phenotypes, highlighting the promise of transdiagnostic and multidimensional approaches to identifying TSRs. The integration of information reflecting adverse and adaptive reactions suggests that psychopathology symptoms and high levels of functioning can coexist, at least in some veterans. This finding runs counter to the notion that TSRs represent a single unitary continuum. Instead, risk and resilience appear to reflect different dimensions that, when integrated, can comprehensively characterize unique TSRs. Future work should continue to investigate the phenotypes identified here as well as other theoretically informed presentations of TSRs to advance research and improve treatment for trauma survivors.

## AUTHOR NOTE

Cameron Pugach is supported by a National Research Service Award (Grant F31MH126528) from the National Institute of Mental Health.

The views expressed in this paper are the authors’ and do not reflect those of the funding agencies, the Department of Veteran Affairs, the U.S. Government, or the authors’ institutional affiliations.

We wish to thank the veterans who participated in the National Health and Resilience in Veterans Study and the Ipsos staff who facilitated data collection.

## OPEN PRACTICES STATEMENT

The study reported in this article was not formally preregistered. Neither the data nor the materials have been made available on a permanent third‐party archive; requests for the data or materials can be sent via email to the senior author at Robert.Pietrzak@yale.edu.

## Supporting information

Supplementary Table S1
