# Supplementary material for: Identifying transdiagnostic traumatic stress reactions in U.S. military veterans: A nationally representative study
Source: J Trauma Stress. 2024 Dec 16;38(2):259–71. doi: 10.1002/jts.23119 (PMC11967292; doi:10.1002/jts.23119)
Supplement: Supplementary file 1 — Supplementary Table S1 [file JTS-38-259-s001.docx]

**Supplementary Table S1**. *Description of Measures/Items Comprising the Protective Psychosocial and Social Connectedness Factors*

| **Protective Psychosocial Characteristics** | | | |
| --- | --- | --- | --- |
| **Construct** | **Measure/Item** | **Citation** | **Internal Consistency (α)** |
| Purpose in Life | Purpose in Life Test-Short Form (4 items) | Schulenberg et al. (2011) | .82 |
| Trait Resilience | Connor-Davidson Resilience Scale-10 (10 items) | Campbell-Stills & Stein (2007) | .92 |
| Trait Grit | Short Grit Scale | Duckworth & Quinn (2009) | .96 |
| Trait Optimism | Life Orientation Test (1 item; “*In uncertain times, I usually expect the best*” | Scheier et al. (1994) | - |
| Trait Gratitude | Gratitude Questionnaire (1 item; “*I have so much in life to be thankful for*”) | McCullough et al. (2002) | - |
| Curiosity/Exploration | Curiosity and Exploration Inventory-II (1 item; “*I frequently find myself looking for new opportunities to grow as a person*”) | Kashdan et al. (2009) | - |
| Community Integration | Perceived level of community integration (1 item; “*I feel well integrated into my community*”) | - | - |
| **Social Connectedness** | | | |
| Perceived Social Support | Medical Outcomes Study Social Support Scale (5 items) | Sherbourne & Stewart (1991) | .91 |
| Attachment Style | Endorsement of secure attachment (Response a) to the following question: “*Please select the statement below that best describes your feelings and attitudes in relationships: (a) feeling that it is easy to get close to others and feelings comfortable with them (secure); (b) feeling uncomfortable being close to others (avoidant); or (c) feeling that others are reluctant to get close (anxious/ambivalent)*.*”* | Hazan & Shaver (1990) | - |
| Structural Social Support | 1 item; “*About how many close friends and relatives do you have [people you feel at ease with and can talk to about what is on your mind]*?” | Sherbourne & Stewart (1991) | - |

*Note.* All single-item measures were rated on a Likert scale ranging from 1 *(strongly disagree)* to 7 *(strongly agree*).

**References**

Campbell-Sills, L., & Stein, M. B. (2007). Psychometric analysis and refinement of the Connor-Davidson Resilience Scale (CD-RISC): Validation of a 10-item measure of resilience. *Journal of Traumatic Stress*, *20*, 1019–1028. https://doi. org/10.1002/jts.20271

Duckworth, A.L., & Quinn, P.D. (2009). Development and validation of the short grit scale (grit-s). *Journal of Personality Assessment*, *91*(2), 166-174. https://doi.org/10.1080/00223890802634290

Hazan, C., & Shaver, P.R. (1990). Love and work: An attachment-theoretical perspective*. Journal of Personality and Social Psychology*, *59*(2), 270-280.

Kashdan, T. B., Gallagher, M. W., Silvia, P. J., Winterstein, B. P., Breen, W. E., Terhar, D., & Steger, M. F. (2009). The curiosity and exploration inventory-II: Development, factor structure, and psychometrics. *Journal of Research in Personality*, *43*(6), 987-998. https://doi.org/10.1016/j.jrp.2009.04.011

McCullough, M.E., Emmons, R.A., & Tsang J.A. (2002). The grateful disposition: a conceptual and empirical topography*. Journal of Personality and Social Psychology*, *82*(1), 112–127. https://doi/10.1037/0022-3514.82.1.112

Scheier, M.F., Carver, C.S., & Bridges, M.W. (1994). Distinguishing optimism from neuroticism (and trait anxiety, self-mastery, and self-esteem): A reevaluation of the Life Orientation Test. *Journal of Personality and Social Psychology*, *67*(6), 1063–1078. https://doi/10.1037/0022-3514.67.6.1063

Schulenberg, S.E., Schnetzer, L.W., & Buchanan E.M. (2011). The purpose in life test-short form: Development and psychometric support. *Journal of Happiness Studies*, *20*, 1–16. https://doi.org/10.1007/s10902-010-9231-9

Sherbourne, C. D., & Stewart, A. L. (1991). The MOS social support survey. *Social Science & Medicine*, *32*(6), 705-714. https://doi.org/10.1016/0277-9536(91)90150-B

**Supplementary Table S2**. *Fit Indices Across Unconditional and Conditional LCA Model Solutions*

| **Model Type** | **Index** | **2 Class** | **3 Class** | **4 Class** | **5 Class** | **6 Class** |
| --- | --- | --- | --- | --- | --- | --- |
| Unconditional | AIC | 44583.18 | 41595.20 | 40340.07 | **39694.36** | 39345.91 |
|  | BIC | 44888.13 | 42055.73 | 40956.18 | **40466.05** | 40273.19 |
|  | SS-BIC | 44732.43 | 41820.59 | 40641.60 | **40072.04** | 39799.74 |
|  | Entropy | .962 | .900 | .915 | **.907** | .893 |
|  | LMR-LRT *p* | <.0001 | <.0001 | <.0001 | **<.0001** | .15 |
|  | BLRT *p* | <.0001 | <.0001 | <.0001 | **<.0001** | <.0001 |
| Conditional | AIC | 124813.42 | 121337.43 | 119841.22 | **118937.37** | 118405.79 |
|  | BIC | 125205.49 | 121916.20 | 120606.70 | **119889.55** | 119544.66 |
|  | SS-BIC | 125005.31 | 121620.70 | 120215.86 | **119403.39** | 118963.18 |
|  | Entropy | .960 | .911 | .920 | **.917** | .903 |
|  | LMR-LRT *p* | <.0001 | <.0001 | <.0001 | **.05** | .12 |
|  | BLRT *p* | <.0001 | <.0001 | <.0001 | **<.0001** | <.0001 |

*Note*. AIC = Akaike Information Criterion; BIC = Bayesian Information Criterion; SS-BIC = Sample Size-Adjusted Bayesian Information Criterion; LMR-LRT *p* = Lo-Mendell-Rubin Likelihood Ratio Test p-value; BLRT *p* = Bootstrapped Likelihood Ratio Test p-value. Unconditional Model included 20 PCL-5 items, 4 PHQ-4 items. Conditional Model included 20 PCL-5 items, 4 PHQ-4 items, 2 functioning items, 1 trauma exposure item, 1 time since trauma item, 1 mental health treatment utilization item.

*Note*. We used an analogous method – latent profile analysis (LPA) – in attempt to model symptom data (i.e., PCL-5, PHQ-4) continuously rather than categorically. The optimal LPA was a 4-class unconditional model that evidenced good fit based on model fit indices (AIC = 155023.68, BIC = 155789.15, SSBIC = 155398.32), entropy (.974), and the LMR-LRT (.016). However, the four classes were only differentiated by symptom severity, not symptom type, meaning that veterans in each class reported the same times of symptoms, only differing by increasing severity. One of these four classes was likely also too small to be generalizable, including only 2.9% of the sample. Thus, compared to the optimal five-class LCA model, where classes were larger and differentiated by both symptom severity and type, the four-class LPA demonstrated smaller classes with less theoretical coherence and clinical utility. Considering these criteria as indicators of an optimal model solution, the five-class LCA model lended more information in differentiating individual traumatic stress reactions.

**Supplementary Table S3**. *Descriptive Statistics for LCA Indicators and Psychosocial Correlates of Class Membership*

| **Variable (Measure)** | ***M* (*SD*) or *n* (%)** | **Range** |  |
| --- | --- | --- | --- |
| **LCA Indicators** |  |  |  |
| PTSD Symptoms (PCL-5) | 2.33 (4.45) | 0-21 |  |
| Depressive Symptoms (PHQ-4) | 0.57 (0.82) | 0-2 |  |
| Anxiety Symptoms (PHQ-4) | 0.53 (0.80) | 0-2 |  |
| Physical Functioning (SF-8 PCS) | 46.81 (9.54) | 12.28-65.76 |  |
| Mental Functioning (SF-8 MCS) | 52.52 (8.33) | 8.17-68.04 |  |
| Total Lifetime Trauma Exposure (LEC-5) | 9.69 (8.50) | 1-60 |  |
| Time Since Index Trauma in Years (LEC-5) | 30.67 (19.64) | 0-87 | |
| Lifetime Mental Health Treatment | 871 (23.5%) | 0, 1 | |
| **Psychosocial Correlates of Class Membership** |  |  |  |
| Protective Psychosocial Characteristics | -0.02 (1.00) | -4.34-2.02 | |
| Social Connectedness | -0.02 (1.00) | -2.23-4.12 |  |
| Loneliness | 4.73 (1.90) | 3-9 |  |

*Note*. PCL-5 = PTSD Checklist for *DSM-5*, PHQ-4 = Patient Health Questionnaire-4, SF-8 PCS and MCS = Short Form-8 Physical Component Scale and Mental Component Scale, LEC-5 = Life Events Checklist for *DSM-5*. PTSD, depressive, and anxiety symptoms reflect the sum of dichotomized items, with PCL-5 items coded as “present” if ≥2 (*Moderately*) and PHQ-4 items coded as “present” if ≥1 (*Several Days*).

**Supplementary Table S4**. *Latent Classifications of PTSD Symptom (PCL-5), Anxiety Symptom (PHQ-4), Depression Symptom (PHQ-4), Physical Functioning (SF-8), Mental Functioning (SF-8), Total Lifetime Trauma Exposure (LEC-5), Time Since Index Trauma Exposure, and Lifetime Psychiatric Treatment History Items and Item Estimates by Class*

|  |  | **Low TSR (*n* = 2400, 64.3%)** | **Avoidant Arousal**  **(*n* = 343, 9.2%)** | **Anxious/**  **Depressive (*n* = 590, 16.6%)** | **Dysphoric Arousal**  **(*n* = 257, 8.2%)** | **High TSR (*n* = 137, 5.7%)** |
| --- | --- | --- | --- | --- | --- | --- |
| **Item** | **Description** | **Est./*M*** | **Est./*M*** | **Est./*M*** | **Est./*M*** | **Est./*M*** |
| PCL1 | Memories | 0.003 | 0.358 | 0.026 | 0.325 | 0.934 |
| PCL2 | Nightmares | 0.001 | 0.212 | 0.008 | 0.167 | 0.797 |
| PCL3 | Flashbacks | 0.001 | 0.108 | 0.005 | 0.131 | 0.726 |
| PCL4 | Psych. Reactions | 0.002 | 0.289 | 0.026 | 0.371 | 0.918 |
| PCL5 | Phys. Reactions | 0.001 | 0.190 | 0.009 | 0.181 | 0.850 |
| PCL6 | Internal Avoidance | 0.008 | 0.490 | 0.025 | 0.402 | 0.932 |
| PCL7 | External Avoidance | 0.006 | 0.420 | 0.018 | 0.388 | 0.908 |
| PCL8 | Amnesia | 0.014 | 0.170 | 0.031 | 0.219 | 0.563 |
| PCL9 | Negative Beliefs | 0.002 | 0.199 | 0.018 | 0.371 | 0.817 |
| PCL10 | Blame | 0.013 | 0.240 | 0.029 | 0.366 | 0.807 |
| PCL11 | Negative Emotions | 0.003 | 0.243 | 0.009 | 0.374 | 0.921 |
| PCL12 | Loss of Interest | 0.011 | 0.199 | 0.085 | 0.486 | 0.852 |
| PCL13 | Distancing | 0.005 | 0.292 | 0.042 | 0.565 | 0.863 |
| PCL14 | Numbing | 0.006 | 0.196 | 0.049 | 0.492 | 0.890 |
| PCL15 | Irritability | 0.003 | 0.150 | 0.035 | 0.349 | 0.805 |
| PCL16 | Risk Taking | 0.002 | 0.074 | 0.013 | 0.168 | 0.410 |
| PCL17 | Hypervigilance | 0.047 | 0.496 | 0.112 | 0.437 | 0.902 |
| PCL18 | Startle | 0.005 | 0.267 | 0.027 | 0.288 | 0.826 |
| PCL19 | Concentration | 0.005 | 0.157 | 0.053 | 0.477 | 0.895 |
| PCL20 | Sleep | 0.058 | 0.439 | 0.156 | 0.625 | 0.872 |
| PHQ1 | General Anxiety | 0.033 | 0.223 | 0.636 | 0.803 | 0.859 |
| PHQ2 | General Worry | 0.025 | 0.174 | 0.558 | 0.753 | 0.797 |
| PHQ3 | Depressed Mood | 0.009 | 0.105 | 0.610 | 0.883 | 0.885 |
| PHQ4 | Anhedonia | 0.046 | 0.193 | 0.628 | 0.859 | 0.897 |
| PCS | Physical Function | 49.17 | 45.71 | 44.50 | 42.56 | 35.82 |
| MCS | Mental Function | 56.79 | 54.27 | 49.13 | 40.27 | 35.19 |
| LEC5 | Lifetime Traumas | 8.30 | 12.32 | 10.93 | 14.56 | 15.18 |
| LEC5 | Time Since Trauma | 35.12 | 32.79 | 31.53 | 28.52 | 29.95 |
| Past Tx | Treatment History | 0.114 | 0.369 | 0.341 | 0.693 | 0.823 |

*Note*. Est. = probability estimates range from 0-1 and indicated probability of item endorsement. *M* = mean scores from assessment measures based on scale construction.

**Supplementary Table S5**. *A Breakdown of Descriptive Statistics for Correlates of Phenotypes Within Each Class*

|  | **Low TSR** | **Anxious/Depressive** | **Avoidant Arousal** | **Dysphoric Arousal** | **High TSR** |
| --- | --- | --- | --- | --- | --- |
| **Demographic Characteristics** |  |  |  |  |  |
| Age | 65.05 (14.33) | 59.05 (16.51) | 60.39 (14.84) | 51.37 (14.40) | 48.69 (13.31) |
| Sex (male) | 2185 (93.1%) | 499 (86.2%) | 264 (82.5%) | 198 (84.8%) | 101 (79.7%) |
| Race (White) | 2033 (81.7%) | 460 (74.4%) | 251 (67.3%) | 204 (76.5%) | 86 (68.6%) |
| Employment (Employed) | 923 (46.4%) | 247 (50.6%) | 157 (53%) | 122 (61.1%) | 54 (45.9%) |
| **Military/Trauma History** |  |  |  |  |  |
| Combat Veteran | 785 (33.1%) | 173 (30.4%) | 140 (46.1%) | 100 (42.1%) | 71 (57%) |
| Index Trauma (non-interpersonal) | 2025 (90%) | 458 (82.3%) | 231 (75.2%) | 164 (77.8%) | 82 (70.7%) |
| **Psychosocial Factors** |  |  |  |  |  |
| Protective Psychosocial Factors | .32 (.78) | -.42 (.92) | -.00 (.88) | -.99 (1.07) | -1.34 (1.11) |
| Social Connectedness | .31 (.86) | -.33 (.93) | -.23 (1.02) | -.97 (.82) | -1.15 (.74) |
| Loneliness | 4.01 (1.43) | 5.44 (1.86) | 5.10 (1.81) | 6.89 (1.73) | 7.21 (1.78) |

*Note*. Weighted estimates were used to compute *M*s, *SD*s, and percentages for each cell. Percentages reflect proportion of veterans within each class.

**Supplementary Table S6**. *A Breakdown of LCA* *Class Membership by Race/Ethnicity*

| Race/Ethnicity | Low TSR | Anxious/Depressive | Avoidant Arousal | Dysphoric Arousal | High TSR |
| --- | --- | --- | --- | --- | --- |
| White, Non-Hispanic | 2033 (81.7%) | 460 (74.5%) | 251 (67.3%) | 204 (76.2%) | 86 (69%) |
| Black, Non-Hispanic | 145 (9.4%) | 43 (11.5%) | 31 (14.3%) | 21 (11.2%) | 19 (15.2%) |
| Hispanic | 148 (5.5%) | 61 (9%) | 44 (12.2%) | 20 (6.6%) | 22 (10.5%) |
| Other, Non-Hispanic | 21 (1.9%) | 9 (3.3%) | 7 (4.5%) | 6 (5%) | 3 (2.3%) |
| Two+ Race, Non-Hispanic | 53 (1.5%) | 17 (1.6%) | 10 (1.8%) | 6 (1%) | 7 (2.9%) |

*Note*. Weighted percentages reflect composition of each LCA class. Post-hoc analyses showed that there was a significant association between race/ethnicity and LCA class, Pearson χ^2^(16) = 78.42, *p* < .001.
